# The investigation of an organic acid assisted sol–gel method for preparing monolithic zirconia aerogels†

**DOI:** 10.1039/c7ra13041d

**Published:** 2018-02-22

**Authors:** Xiaoqing Wang, Chengyuan Li, Zhenyu Shi, Mingjia Zhi, Zhanglian Hong

**Affiliations:** State Key Laboratory of Silicon Materials, School of Materials Science and Engineering, Zhejiang University Hangzhou 310027 China mingjia_zhi@zju.edu.cn hong_zhanglian@zju.edu.cn

## Abstract

In our previous work, a citric acid assisted sol–gel method was developed for preparing monolithic metal oxide aerogels. Such method adopted citric acid as the gelator, which replaced the well-studied proton scavenger propylene oxide. In this work, we have further extended this “organic acid assisted” sol–gel method and investigated the gelation mechanism. Four different organic acids (butanedioic acid, l-malic acid, l-aspartic acid and mercaptosuccinic acid) with an identical main chain but different side groups were used as the gelators for preparing monolithic zirconia aerogels. It was found that complex interactions including covalent bond and coordination bond interactions between organic acids and zirconium ions were vital to give a rigid gel network. After supercritical drying, crystalline zirconia aerogels can be obtained with high surface areas over 330 m^2^ g^−1^ and large pore volumes over 3.574 cm^3^ g^−1^.

## Abstract 2

The mechanism of the organic acid assisted sol–gel method free of propylene oxide for preparing monolithic zirconia aerogels was investigated in detail.

## Graphical abstract

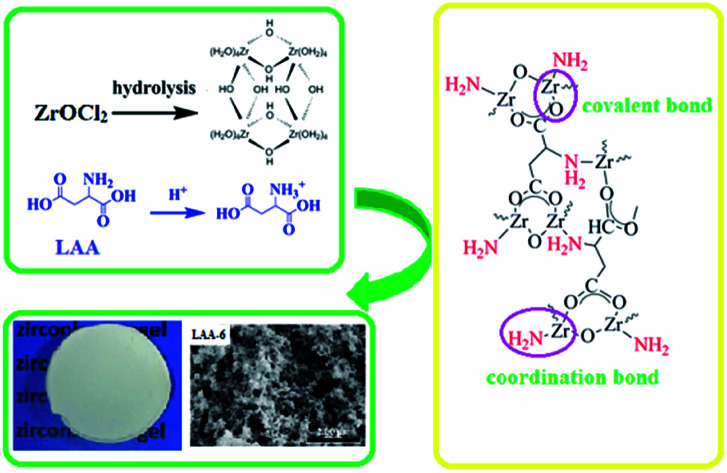

## Introduction

The ZrO_2_ aerogel has attracted lots of interest due to its potential applications as catalyst supports,^1,2^ absorbents,^3–5^ light-weight thermal insulators^6,7^*etc.* In the literature, two well established methods have been developed to prepare ZrO_2_ aerogels, namely “alkoxide hydrolysis” and “epoxide adding” methods. The first one relies on the controllable hydrolysis of organic zirconium alkoxide, and the later one adopts inorganic zirconium salts and propylene epoxide as reagents. When alkoxides are used as precursors, the fast hydrolysis rate of Zr^4+^ often prevents the formation of a well-developed gel network and gives a precipitate instead. In addition, alkoxides are usually sensitive towards environmental humidity. Thus the “epoxide adding” method is preferred now since it is applicable to prepare wet gels from multiple precursors such as oxynitrates, oxychlorides, and chlorides. In this method, propylene epoxide (PO) is taken as the proton scavenger.^8–14^ For instance, Guo and coworkers have successfully prepared well-defined macroporous yttria-stabilized zirconia (YSZ) monoliths by applying zirconium oxychloride (ZrOCl_2_·8H_2_O) as the precursor and PO as the gel accelerator.^8^ Crack-free monolithic ZrO_2_ aerogel modified by SiO_2_ (ZSA) has also been prepared by similar reagents.^12^ Hu's group has reported that translucent monolithic zirconia aerogels with a high specific surface area of 454 m^2^ g^−1^ can be obtained in the presence of the PO in both ethanol and mixed ethanol–water solutions.^13^ Gash's group has reported that homogeneous, nanocrystalline YSZ aerogel can be obtained using the epoxide addition sol–gel method.^14^ Such method is also applicable to prepare other metal oxide aerogels. Some representative examples include alumina oxide,^15^ ferric oxide,^16,17^ nickel oxide,^18^*etc.*^19^ However, PO has the drawbacks such as toxicity, inflammability and explosiveness, which may limit the scale-up synthesis of metal oxide aerogels.

In responding to the above issues, our group has recently developed an environmental friendly sol–gel method for preparing various metal oxide aerogels, including zirconia and nickel oxide aerogel.^20,21^ In such method, an organic acid (citric acid) was key to initiate the sol–gel transition. With the aid of citric acid, ZrOCl_2_·8H_2_O and Ni(NO_3_)_2_·6H_2_O solution can turn to strong wet gel, which was the pivotal step in sol–gel process. Monolithic metal oxide can be obtained after supercritical drying the wet gel. This method overcame the drawbacks of epoxide addition method since the chemicals were much less harmful. It is believed that the interactions among the hydroxyl groups (–OH, in citric acid) play significant roles in crosslinking the metal ions to form the network. The acidic environment also slows down the hydrolysis rate, which prevents precipitation. As a result, rigid gel skeleton could form.

However, the mechanism of the gelation process is still in mist. For example, the detailed interactions between the organic acid and the metal ions, the influence of the molecular structure of the acid, as well as the relative reagents ratio could all affect the gelation process. It is then vital to further understand the gelation mechanism, which will enable the further improvement and generalization of this approach.

Based on the above discussion, in this work, we rationally selected four organic acids, namely l-aspartic acid, butanedioic acid, l-malic acid, and mercaptosuccinic acid as the gelators to prepare zirconia aerogels. These acids possess identical main chain while being different in their side groups (–NH_2_, –H, –OH and –SH), which offer suitable research objects to further understand the gelation mechanism. The role of such side groups is highlighted by the different electronegativity of oxygen (3.5), nitrogen (3.0) and sulfur (2.5) atoms, leading to the greater interaction between hydroxyl and zirconium ion (Zr^4+^–OH) than that of Zr^4+^–NH_2_ or Zr^4+^–SH. Moreover, the radius and the number of nuclear charges of sulfur are larger than those of oxygen and nitrogen. Their influence of such differences on the wet gel formation were studied and analyzed. This work shed light to further in-depth interpretation of the mechanism of wet gel formation.

## Experimental

### Materials

All chemical reagents were analytically pure and were used as received without further purification, including zirconium oxychloride (ZrOCl_2_·8H_2_O, 99.9%, Aladdin, China), l-aspartic acid (LAA, C_4_H_7_O_4_N, 99%, Energy Chemical, China), mercaptosuccinic acid (MSA, C_4_H_6_O_4_S, 98%, Energy Chemical, China), l-malic acid (LMA, C_4_H_6_O_5_, 98%, Aladdin, China), butanedioic acid (BA, Sinopharm Chemical Reagent Co., Ltd (SCRC)), hydrochloric acid (HCl, Sinopharm Chemical Reagent Co., Ltd (SCRC)) and ethanol absolute (EtOH, SCRC).

### Characterization

The morphology of the aerogels was observed by using HITACHI S-4800 scanning electron microscope (SEM) and transmission electron microscope (TEM: JEM-1200EX, JEOL, Japan). X-ray photoelectron spectroscopy (XPS) was carried out in Escalab 250Xi instrument. X-ray diffraction (XRD) measurements were performed in a X-ray diffractometer (X'Pert PRO, PANalytical B.V.) with the use of Cu K_α_ radiation (*λ* = 1.5418 Å) at 4° min^−1^ scanning speed in the 2*θ* range from 10–90°. Thermogravimetric analysis (TGA) and differential thermal analysis (DTA) were performed on a TA-Q 500 TGA instrument. Samples were pretreated at 100 °C for 30 minutes, and then heated to 1000 °C at a rate of 10 °C min^−1^ in air. Fourier Transform Infrared Spectroscopy (FT-IR) was recorded on Nicolet 5700 spectrophotometer using KBr pellets containing 1% weight sample in KBr. The nitrogen adsorption–desorption measurements (Quantachrome Instrument Corp) were used to obtain the nitrogen physisorption isotherms at 77 K. Surface areas were evaluated using the Brunauer–Emmett–Teller (BET) method from the adsorption branch of the isotherm. The pore size distributions were calculated according to the Barrett–Joyner–Halenda (BJH) model, and the average pore diameters and cumulative pore volumes were calculated using the desorption branch of the isotherm.

### Preparation of zirconia wet gel

The wet gel was prepared by mixing ZrOCl_2_·8H_2_O and organic acid. Taking LAA as an example, in a typical preparation, ZrOCl_2_·8H_2_O (8.89 g, 27.6 mmol) was completely dissolved in 60 mL ethanol and stirred to give colorless and transparent solution. A given volume (2–10 mL) of LAA (0.8 mol L^−1^) in HCl/ethanol solution (v/v = 1/4) was then quickly added into the above solution under continuous stirring. The sol was then transferred to sealed glass culture dishes preheated at 60 °C, and wet gels were formed when the sols no longer flow when the glass culture dishes were tilted. The resulted wet gels were aged for 2 h at 60 °C and then for 48 h at 40 °C in ethanol in the closed containers. Deserved to be mentioned, BA, LMA and MSA can be dissolved in ethanol directly, and the concentration of the BA, LMA and MSA ethanol solution was 1 mol L^−1^. The organic acid solutions were then mixed with ZrOCl_2_·8H_2_O in a similar manner to give the wet gels.

**Scheme 1:**
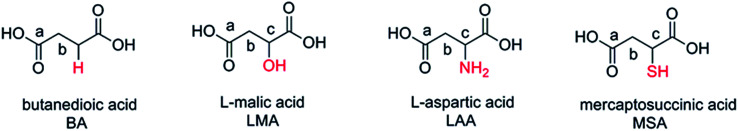
The structural formulae of organic acids BA, LMA, LAA and MSA.

The structural formulae of organic acids BA, LMA, LAA and MSA were presented in Scheme 1, and the experimental parameters for the as prepared ZrO_2_ xerogels and aerogels were listed in Table 1. The samples in Table 1 were grouped according to the organic acids used in the experiments, such as LAA, LMA, MSA and BA series. Since the volume of ZrOCl_2_·8H_2_O solution was fixed in the experiments (60 mL), the samples were named by the abbreviation of the organic acid followed by the volume number of the organic acids solution used. For example, LAA-2 stood for the sample prepared by adding 2 mL of LAA solution into 60 mL of ZrOCl_2_·8H_2_O solution. In additional, control experiment was carried out to prepare ZrO_2_ aerogels by mixing 60 mL of ZrOCl_2_·8H_2_O solution with 8 mL of PO, and the sample was labeled as PO-8 series.

**Table 1:** The experimental parameters of the preparation of ZrO_2_ aerogels and xerogels

Sample series	Acid solution (mL)	Gelation time (min)	Color of the gel	Gelation temperature (°C)	Xerogel	Aerogel
BA-4^a^	4	—b	—	60	—	—
BA-6	6	—	—	60	—	—
LAA-2^c^	2	80	White	60	✓	✓
LAA-4	4	10	White	60	✓	✓
LAA-6	6	7	White	60	✓	✓
LAA-7.5	7.5	1	White	60	✓	✓
LAA-8	8	1	White	60	✓	✓
LAA-10	10	<0.5	White	60	✓	✓
LMA-4^d^	4	1.5	White	60	✓	✓
LMA-6	6	<0.5	White	60	✓	✓
LMA-8	8	Immediately	White	60	✓	✓
MSA-6^e^	6	24	White	60	✓	✓
MSA-8	8	15	White	60	✓	✓

a BA-4 means the volume of the gelation accelerator is 4 mL of BA.

b — means there was no wet gel formation, only precipitation.

c LAA-2 means the volume of the gelation accelerator is 2 mL of LAA.

d LMA-4 means the volume of the gelation accelerator is 4 mL of LMA.

e MSA-6 means the volume of the gelation accelerator is 6 mL of MSA.

### Preparation of zirconia aerogel and xerogel

ZrO_2_ aerogels were obtained by supercritical drying the wet gels in ethanol (temperature: 260 °C, pressure: 7 MPa). In a typical drying process, 300 mL of ethanol and wet gel of the volume of ∼20 mL were loaded into an autoclave. The ethanol volume was much excess to the wet gel volume. ZrO_2_ xerogels were obtained by directly drying the wet gels at 60 °C in atmospheric pressure. It should be noted that in this study, xerogels were used as approximation to the wet gels, since they were directly dried from wet gel under mild conditions.

## Results and discussion

Fig. S1† gives the typical appearances of the wet gel and the aerogel. Fig. S1(a) and (b)† shows the photos of LAA-4 wet gel and LAA-4-aerogel, respectively. It can be seen that the monolithic appearance of the wet gel can be well reserved in the aerogel. This is due to the fact that the supercritical drying process prevented the pores collapse in the wet gel during the drying. During drying, the majority of the organic acid can also be removed since they are soluble in ethanol, which will be verified by TGA and XPS later. Fig. S1(c) and (d)† showed the photos from LMA-6 series, and similar result can be observed. This indicated that the method developed here can prepare monolithic ZrO_2_ aerogel.

The gel formation mechanism of different organic acids was firstly verified by directly observing the sol–gel transition (when the sol does not flow) after ZrOCl_2_·8H_2_O and the organic acid were mixed, and the gelation time was also recorded. Interestingly, it was found that wet gels could easily form when LMA, LAA and MSA were applied as the gelators. In stark contrast to that, no monolithic wet gel formed when BA was adopted, even the experimental parameters (temperature, time, molar ratio, pH, *etc.*) were adjusted for several batches. From Scheme 1, it can be seen that the only difference between the above organic acids is the side group. BA has no side group, while LMA, LAA and MSA have side groups of –OH, –NH_2_ and –SH, respectively. Thus, these side groups in organic acids are vital in the gelation process, since complex interactions between the organic acid and Zr^4+^ ions can be formed, such as coordination bond interaction and hydrogen bond interaction.

The side group effect was further confirmed by comparing the different gelation time needed for LMA, LAA and MSA. From Table 1, one can see that the amount of organic acid had great impact on the gel formation rate, since the gelation time decreased with the increase of the amount of LMA/LAA/MSA. However, in comparison with its MSA-6 (MSA-8) and LAA-7.5 (LAA-10) counterparts, LMA-6 (LMA-8) showed the fastest gelation rate, whilst MSA-6 and MSA-8 required longest time to complete the gelation. Such discrepancy perfectly coincided with the electronegativity sequence of oxygen (3.5), nitrogen (3.0) and sulfur (2.5). In the framework of HSAB theory, MSA (R–SH) is soft base (SB), whereas LMA (R–OH) and LAA (R–NH_3_^+^) are hard bases (HBs).^22^ Given the fact that zirconium ion is hard acid (HA), it interacts with LMA and LAA more easily. Another point that should be noted is that the radius and the number of nuclear charges of sulfur are larger than those of oxygen and nitrogen, and this may lead to the slower gelation rate of MSA-6(8).

**Fig. 1:**
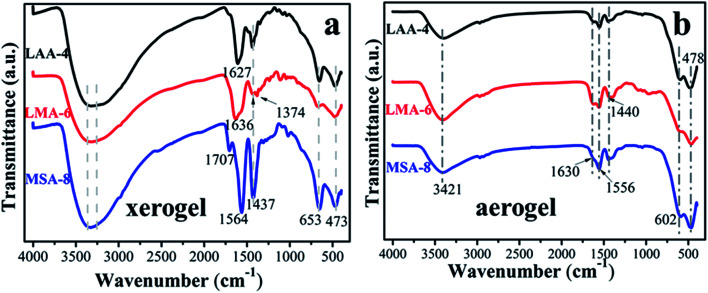
FT**-**IR spectra of (a) ZrO_2_ xerogels and (b) aerogels with different organic acids.

In order to further investigate the gelation mechanism, FT-IR spectra of the three typical xerogels and aerogels samples series (LAA-4, LMA-6 and MSA-8) were analyzed as shown in Fig. 1. The broad bands from 3260 cm^−1^ to 3360 cm^−1^ in Fig. 1(a) were attributed to the –OH vibration of the samples and the adsorbed water on their surface.^8,23,24^ The narrow bands situated at 653 cm^−1^ and 473 cm^−1^ were attributed to the Zr–O bond, in line with the following XRD results. The absorbance of *ν*_as_(C=O) vibration in LMA-6 was located at 1636 cm^−1^, while in LAA-4 and MSA-8 they were at 1627 cm^−1^ and 1564 cm^−1^, respectively.^25^ Compared with carboxyl in raw organic compounds (1760–1660 cm^−1^), this peak exhibited obvious red-shift, which was ascribed to the covalent bond between Zr^4+^ ion and carboxyl, and was also rooted in the fact that there was hydrogen bond interaction between the O atom in C=O and the H in –OH, –NH_2_ and –SH. In addition, the O in –OH in LMA-6 had the largest electronegativity, which led to the largest ability of electron-withdrawing, resulting in the largest infrared absorption wavenumber. The band at 1437 cm^−1^ was assigned to *ν*_s_(C–O) vibration.^26–28^ In additional, very weak or no C=O stretch band of carboxyl group in original organic acid (typically at 1710 cm^−1^) was seen in the spectra. Furthermore, the interaction between the carboxylate head and the metal atom can be categorized as four types: monodentate, bridging bidentate, chelating bidentate, and ionic interaction.^29–31^ The wavenumber separation *Δ* between the *ν*_as_(C=O) and *ν*_s_(C–O) IR bands, can be used to diagnose the type of the interaction between the carboxylate head and the metal ion. In this work, the *Δ* (110–200 cm^−1^) was ascribed to the bridging bidentate, where the interaction between the COO^−^ group and the Zr^4+^ ion was covalent.^32^ For the aerogel samples, it can be seen that there was barely no difference between the samples in Fig. 1(b). This is because that most organic compounds have been removed during the supercritical drying process, and leaving pristine ZrO_2_ aerogel. The peaks located at 402 and 678 cm^−1^ can be assigned to Zr–O bond.

Esterification like reactions may occur during the sol–gel transition. Here such reactions can be well excluded due to the following reasons. Firstly, high temperature and catalyst were required for esterification, which were absent in the experiment (the sol–gel transition temperature was 60 °C and there was no catalyst). Secondly, the FTIR spectra of LAA-4-xerogel showed that there were neither signs of carbonyl (–O=C–NH–) stretching vibration band at 1670–1650 cm^−1^, nor amide II and amide III related vibration bands at 1560–1520 cm^−1^ and 1240 cm^−1^, respectively. The magnified FTIR spectra of LAA-4-xerogel at these ranges can be found in Fig. S2.† Similarly, there was no carbonyl (–O=C–S–) stretching vibration at 1740 cm^−1^ in MSA-8-xerogel, as shown in Fig. S3.† In addition, no peaks related to C=O linked C–S was found at about 1240–1150 cm^−1^.^33^ Therefore, the possible esterification can be neglected.

**Scheme 2:**
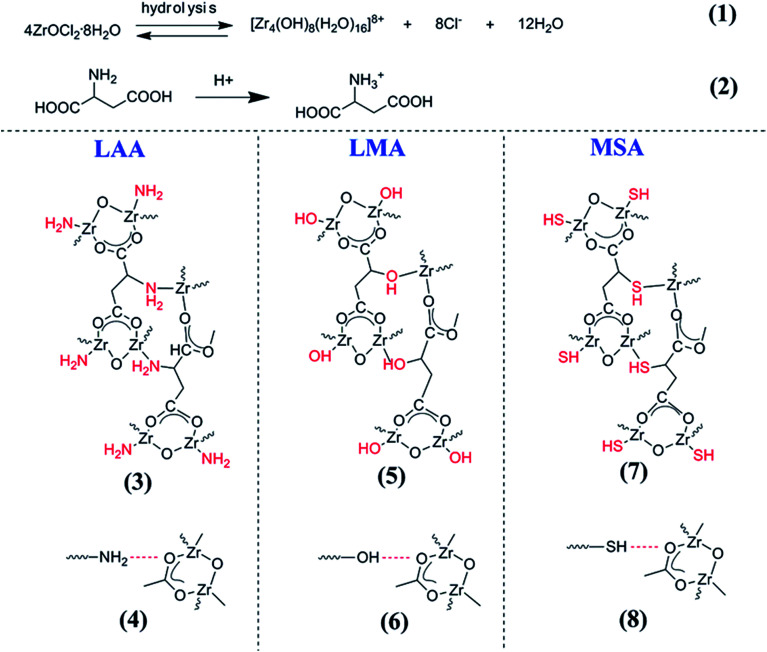
The schematic of the reaction routes between the organic acid and the Zr ions, which reveals the gel formation process.

Based on the above observation, one may propose the gelation reactions. Scheme 2 listed the possible reaction pathways. It has been reported that ZrOCl_2_·8H_2_O in ethanol solution could hydrolyze to [Zr_4_(OH)_8_·(H_2_O)_16_]^8+^ complex cluster as shown in reaction (1).^34,35^ Taking LAA as an example, such complex clusters would have multiple interactions with LAA. One possible interaction was that the zirconium ions (Zr^4+^) in [Zr_4_(OH)_8_·(H_2_O)_16_]^8+^ complex cluster would combine with carboxyl group (O^−^–C=O) by the bridging bidentate, and another integration was combining with amino group (–NH_2_) in LAA, which was written in the reaction (3) in Scheme 2. The later one was strong coordination bond. In the meantime, the carboxyl group in LAA would probably interacts with –NH_2_ (in reaction (4)), which was the hydrogen bond interaction. Such complex bond interactions expanded the cross-lined network in three dimensions to form gel skeleton. The same mechanism also worked in the case of LMA and MSA series samples, as the Zr^4+^ ions would coordinate to –OH and –SH in the side groups (as shown in reaction (5) and (7)), when such –OH and –SH would also form hydrogen bond (in reaction (6) and (8)) to extend the network. As a result, rigid complex molecules backbone can form. This mechanism also explained the different gelation time between the organic acids. Since the coordination bond brought by the side groups played important role in the gel network formation, the gelation time followed the order of the capability to form coordination bond with Zr^4+^ (in the order of O > N > S, and the gelation rate was in the order of LMA > LAA > MSA). The above mechanism can also well explain the gelation process using citric acid as gelator in our previous papers, since citric acid owned the similar structure with side group of –OH. On the other side, since there is no additional side group in BA, no such coordination bond could form between BA and Zr^4+^ ions, as well as the hydrogen bond among BAs, thus no gel could form.

**Fig. 2:**
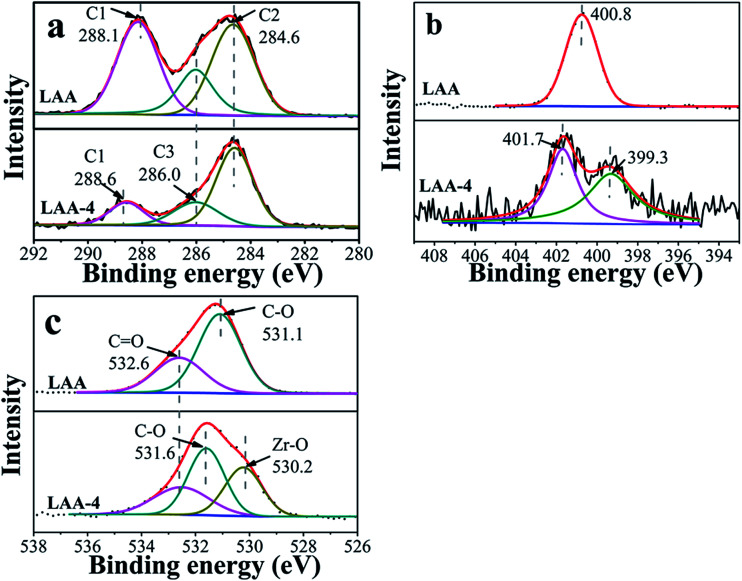
XPS spectra of C 1s (a), N 1s (b) and O 1s (c) in monomer LAA and xerogel LAA-4.

XPS was further used to illustrate interactions enabling the formation of ZrO_2_ gel. Here XPS spectra of the original organic acids, the xerogels (directly dried wet gel) and the aerogels were taken and compared. Fig. S4 and S5† showed the results of C 1s, N 1s/S 2p and O 1s core levels of LAA-4 and MSA-8 series aerogels. There was no N 1s signal in LAA-4-aerogel (seen in Fig. S4(c)†), and the signal of S 2p in MAS-8-aerogel was very weak, which was hardly distinguished (seen in Fig. S5(b)†). This confirmed that the organic acid can be effectively removed using the drying parameters. Fig. 2 showed the comparison of C 1s, N 1s and O 1s core levels of LAA and LAA series xerogels. C 1s was deconvoluted into three peaks as in Fig. 2(a), which can be attributed to C1 (HO–C=O), C2 (HC–CH_2_) and C3 (HC–OH) separately, and the detailed binding energy of them were presented in Table 2. The peak at 284.6 eV was the aliphatic chain (C–C), the peak at 285.9 eV and 288.1 eV were attributed to C–O and HO–C=O, respectively. However, in LAA-4-xerogel, the relative intensity of the carboxylic acid carbon related peak reduced significantly to that of LAA. Such observation matched with the proposed mechanism discussed above, as the carboxylic acid end of the LAA would bond to Zr^4+^ ions. As result, the peak at 288.6 eV emerged, which was corresponding to the carboxylate. The same trend was also observed in the C 1s spectra in LMA and MSA series, indicating the similar mechanism. The N 1s spectra were plotted in Fig. 2(b). It can be seen that the N 1s peak in LAA was located at 400.8 eV and showed obvious shift in LAA-4 xerogel. There were two peaks located at 399.3 eV and 401.7 eV in the xerogel, which was due to the complex coordination interactions of N and zirconium ion and hydrogen bond interaction of N and H. Similarly, as presented in Fig. 2(c), O 1s spectra of LAA was deconvoluted into two peaks, which were attributed to the oxygen in C–O and C=O respectively. In the xerogel, a new O 1s peak emerged at about 530.2 eV, which could be attributed to O in the oxide lattice.^36–38^

**Table 2:** The binding energy of C, O, N/S in organic acids and aerogel and xerogel samples

Sample series	Binding energy of C (eV)			Binding energy of O (eV)			Binding energy of N/S (eV)	
	**–**CH_2_	–CH–X	–COOH	Zr–O	C–O	C=O		
LAA^a^	284.6	285.9	288.1	—	531.1	532.6	400.8	
LAA-4-xerogel	284.6	286.0	288.6	530.2	531.6	532.5	399.3	401.7
LAA-4-aerogel	284.6	286.0	288.5	530.1	531.7	533.1	No signal	
LMA^b^	284.6	286.3	288.6		531.9	532.8	—	
LMA-6-xerogel	284.6	286.2	288.8	530.5	531.8	533.1	—	
MSA^c^	284.6	—	288.4	—	531.7	532.9	162.6	163.8
MSA-8-xerogel	284.6	285.6	288.6	530.4	531.8	533.3	163.1	164.3
MSA-8-aerogel	284.6	286.0	288.5	530.1	531.7	533.1	163.3	164.8
PO-8-xerogel	284.6	287.4	—	529.5	530.9	—	—	—
PO-8-aerogel	284.6	287.4	—	529.6	531.2	—	—	—

a CHX means CHOH in LMA.

b CHX means CHNH_2_ in LAA.

c CHX means CHSH in MSA.

The corresponding XPS spectra for LMA and MSA series xerogels can be found in the ESI Fig. S6 and S7.† In additional, Fig. S8† showed the C 1s and O 1s core levels of aerogel PO-8-aerogel, and Fig. S9† showed the comparison of Zr 3d in LAA-4-aerogel and PO-8-aerogel. A detailed peak assignment was summarized in Table 2. From Table 2, it was found that the peak shifting in three xerogel samples held the similar trends. The intensity of the carboxylic acid peak in C 1s decreased in the xerogel samples, while carboxylate related peak at higher binding energy appeared. In the meantime, the lattice oxygen can be always found in the xerogel samples, indicating the formation of Zr–O bond. The C 1s in PO-8-aerogel was deconvoluted into two peaks which can be attributed to CH_2_–CH_2_ and H_2_C–O separately. The peak at 284.6 eV was the aliphatic chain (C–C), and the peak at 287.4 eV was attributed to H_2_C–O. Similarly, O 1s spectra of PO-8-aerogel was deconvoluted into two peaks, which were attributed to the oxygen in CH_2_O and Zr–O respectively. It can be seen that the Zr 3d_5/2_ and 3d_3/2_ in LAA-4-aerogel were 0.7 eV larger than that in PO-8-aerogel, which was attributed to the –COO– linked with Zr in the aerogel LAA-4-aerogel.

The above analysis of FT-IR and XPS data clearly revealed the gel formation mechanism assisted by organic acid. A suitable organic acid molecule structure, which contained carboxylic acid ends and side groups such as –OH, –NH_2_ and –SH *etc.*, was required for successful formation of the gel backbone. Especially, the coordination bonds between Zr^4+^ ion and the side group were vital to extend the gel network.

**Fig. 3:**
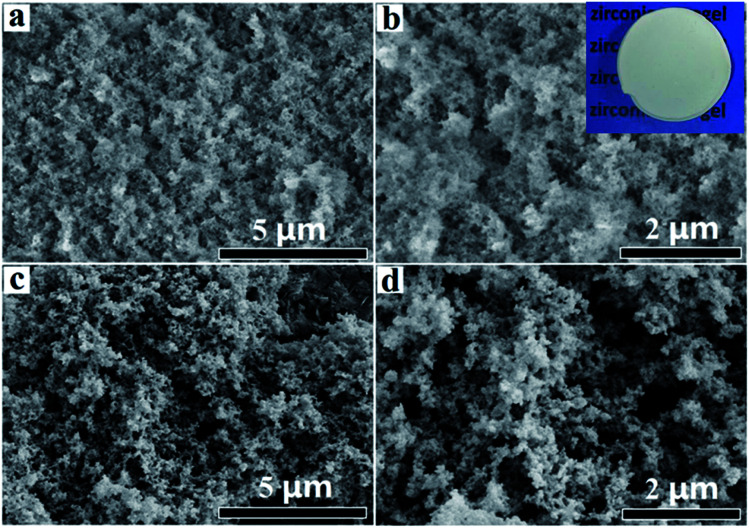
(a) and (b) SEM images of LAA-4 aerogel, (c) and (d) LAA-6 aerogel.

**Fig. 4:**
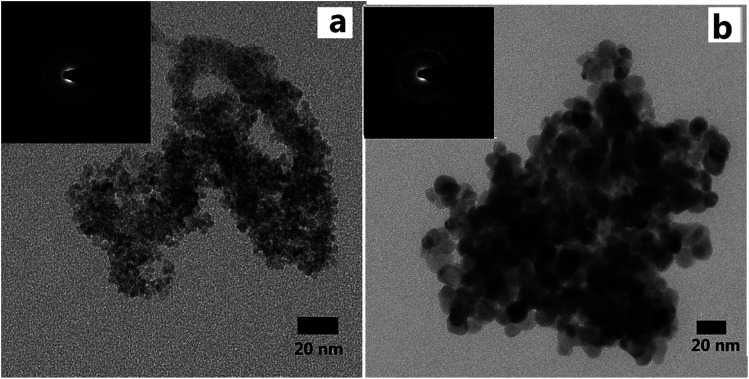
TEM images of LAA-6 aerogel as-prepared (a) and after heat treatment at 600 °C (b), and the insets show the selected area electron diffraction pattern.

Aerogels were obtained after supercritical drying. Fig. 3 showed the SEM images of LAA-4 and LAA-6 aerogels. It was obvious that the aerogels were aggregated by numbers of nanoparticles, and most of the pores were mesopores (Fig. 3). The morphology was similar to those prepared from “alkoxide hydrolysis”^39^ and “epoxide adding”^40^ methods, in which the aerogel was composed of porous networks. The insert image in Fig. 3(b) showed that monolithic aerogel can be obtained by such method. A further investigation of aerogel microstructure was achieved by TEM, which revealed the particle size in the aerogel. Fig. 4 showed the TEM images of the as-prepared LAA-6-aerogel and the sample after calcination at 600 °C. From Fig. 4(a), it can be seen that before calcination, most of the particles were agglomerated to clusters and the boundaries of the particles were not clearly identified. Numerous pores can also be identified between the particles, revealing the porous nature of the aerogel. After calcinations at 600 °C, the particles size was about 15 nm as shown in Fig. 4(b). The SAED patterns in Fig. 4 showed diffraction rings, which indicated the aerogels were crystallized after supercritical drying. This was in accordance with the XRD results discussed later. Fig. S10† also showed the TEM images of the as prepared MSA-8-aerogel and the sample after calcination at 1000 °C. The particles in Fig. S10(a)† were similar to that in Fig. 4(a), while the particles were agglomerated to larger ones after calcination at higher temperature, as shown in Fig. S10(b).† This may be attributed to the heat-labile of the ZrO_2_ aerogel.

**Fig. 5:**
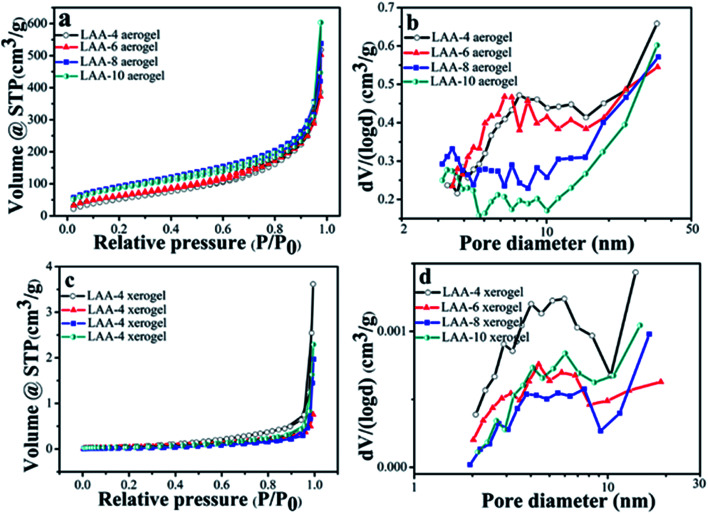
Nitrogen adsorption–desorption isotherms for the aerogel and xerogel samples LAA-4, LAA-6, LAA-8 and LAA-10 (a and c), and their pore size distributions (b and d).

The surface area of the aerogels and the xerogels was examined by N_2_ adsorption–desorption. Fig. 5(a) and (c) were the isothermal curves for the aerogels and the xerogels using LAA as the gelator. The aerogels showed similar type II behavior, indicating macropores existed in the samples. The surface area and the pore volume were summarized in Table 3. From Table 3, we can see that LAA-4, LAA-6, LAA-8 and LAA-10 aerogels had the surface area of 221, 236, 330 and 315 m^2^ g^−1^, and the pore volume of 1.676, 1.811, 2.152 and 3.574 cm^3^ g^−1^, respectively. In contrast, all LAA series xerogel samples had the surface area lower than 0.15 m^2^ g^−1^, and the pore volume lower than 0.006 cm^3^ g^−1^, attributing to the collapse of the pores in the process of ambient pressure drying. The isothermal curves can also be attributed to non-porous materials. The analysis of the mesopore size distribution was accomplished by BJH method, as was shown in Fig. 5(b) and (d). The majority size of the mesopores for all the samples was below 50 nm, which was similar with other zirconia aerogels reported before. For examples, the surface area of zirconia aerogels prepared by zirconium *n*-propoxide *via* sol–gel method was 142 m^2^ g,^41^ and the surface area of the aerogel was 250 ± 37 m^2^ g^−1^ by adding HNO_3_ into zirconium *n*-propoxide solution.^42^ This proved that the ZrO_2_ aerogel prepared by the organic acid assisted method was analogy to those prepared by the alkoxide hydrolysis method. For the pore size distributions, it was found that dispersive peaks below 10 nm were found in LAA-4-aerogel and LAA-6 aerogel. In LAA-8-aerogel and LAA-10 aerogel samples, bigger pore size distribution was observed and the major pore size were larger than 30 nm (beyond the calculation limit of BJH method). This may result from the decomposition of the excess LAA during the supercritical drying process.

**Table 3:** The surface area, pore volume of the LAA aerogel and LAA xerogel series samples

Sample series	Surface area (m^2^ g^−1^)	Pore volume (cm^3^ g^−1^)
LAA-4 aerogel	221	1.676
LAA-4 xerogel	0.13	0.006
LAA-6 aerogel	236	1.811
LAA-6 xerogel	0.12	0.001
LAA-8 aerogel	330	2.152
LAA-8 xerogel	0.05	0.003
LAA-10 aerogel	315	3.574
LAA-10 xerogel	0.13	0.004

**Fig. 6:**
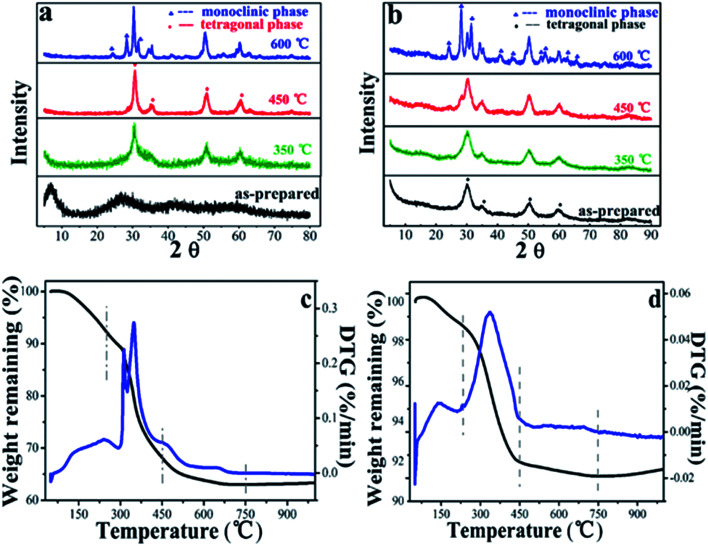
XRD spectra of (a) LAA-4-xerogel and (b) aerogel, for the as prepared sample and those treated with 350 °C, 450 °C and 600 °C, (c) and (d) are temperature-dependent weight remaining and the corresponding derivatives of LAA-4 xerogel and aerogel.

The XRD pattern of LAA-4 xerogel and aerogel were shown in Fig. 6(a) and (b).

For the as prepared samples, it was found that the xerogel was amorphous and the aerogel was crystalized. The as-prepared aerogel had broad diffraction peaks at 30.2°, 35.2°, 50.3° and 60.2°, which can be indexed as tetragonal phase (JCPDS card no. 50-1089). Such tetragonal phase was stable after heating at 350 °C. When the heating temperature was raised to 450 °C, monoclinic phase appeared, and the aerogel after heating at 600 °C showed mixed phase.^13,43^ The xerogels samples showed similar trend when heated at 350 °C, 450 °C and 600 °C. This was consistent with the TGA/DTA results as shown in Fig. 6(c) and (d). It can be seen that in the first stage (50–250 °C), the initial 8% (xerogel) and 1% (aerogel) of the weight loss were assigned to the weakly absorbed water and the dehydroxylation of zirconium hydroxide. In the second stage (250–450 °C), the relatively high weight loss of 24 wt% (xerogel) and 5 wt% (aerogel) were corresponding to the partial decomposition of the residue LAA (Fig. S11,† TGA and DTA curves of LAA) and ZrO_2_ phase transition. This can be further proved by the XRD spectra of sample LAA-4-xerogel and aerogel treated at 450 °C in Fig. 6.^39^ In the third stage, there was about 5% (xerogel) and 1% (aerogel) weight loss from 450 to 700 °C, attributing to the complete decomposition of LAA and further crystalline phase transferring of ZrO_2_. The weight loss of aerogels was much less than that of its corresponding xerogel samples, which was consistent with the former FTIR and XPS results.

## Conclusions

In summary, the gel formation mechanism of the newly proposed “organic acid assisted” sol–gel method for preparing ZrO_2_ aerogel was revealed by employing four different kinds of organic acids (butanedioic acid, l-aspartic acid, l-malic acid and mercaptosuccinic acid) as the gelator. The complex interactions between the organic acids and Zr^4+^ ions were thoroughly investigated, including covalent bond and coordination bond interactions, which yielded robust gel network. Crystallized monolithic ZrO_2_ aerogel was obtained after supercritical drying, and showed characteristics of high surface area and large pore volume.

## Conflicts of interest

There are no conflicts to declare.

## Supplementary Material

RA-008-C7RA13041D-s001
