# Supplementary material for: The investigation of an organic acid assisted sol–gel method for preparing monolithic zirconia aerogels
Source: RSC Adv. 2018 Feb 22;8(15):8011–20. doi: 10.1039/c7ra13041d (PMC9078517; doi:10.1039/c7ra13041d)
Supplement: RA-008-C7RA13041D-s001 [file RA-008-C7RA13041D-s001.pdf]

Electronic Supplementary Material (ESI) for RSC Advances.
This journal is © The Royal Society of Chemistry 2018

The investigation of organic acids assisted sol-gel method for preparing monolithic zirconia aerogels

Xiaoqing Wang[a], Chengyuan Li[a], Zhenyu Shi[a], Mingjia Zhi[a]* and Zhanglian Hong[a]*

State Key Laboratory of Silicon Materials, School of Materials Science and Engineering, Zhejiang University, Hangzhou 310027, China.

* Corresponding author: mingjia_zhi@zju.edu.cn; hong_zhanglian@zju.edu.cn.

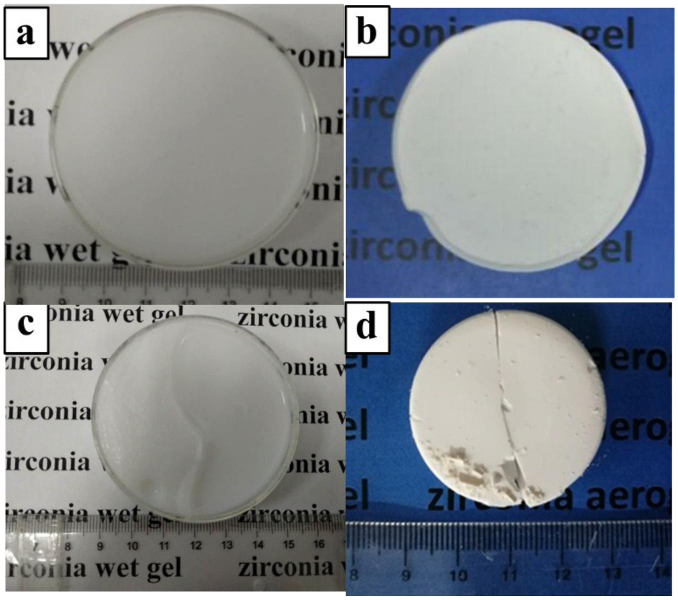

Figure S1 Photos for the wet gel and the aerogel after supercritical drying, (a) LAA-4-wet gel, (b) LAA-4-aerogel, (c) LMA-6-wet gel and (d) LMA-6-aerogel.

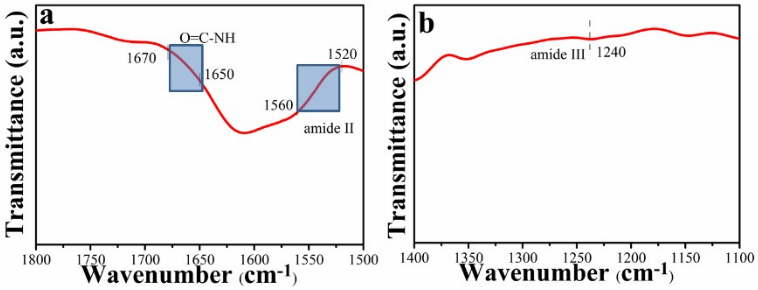

Figure S2 Enlarged FT-IR spectra of LAA-4-xerogel from1800-1500 cm^{-1} and 1400-1100 cm^{-1}, and the possible peak positions for O=C-NH, amide II and amide III vibration bands are marked.

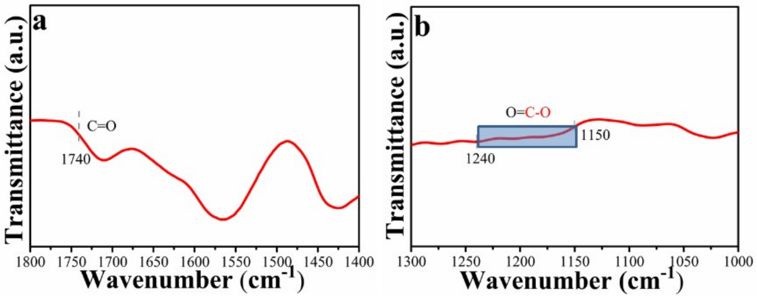

Figure S3 Enlarged FT-IR spectra of MSA-8-aerogel from1800-1400 cm^{-1} and 1300-1000 cm^{-1}, and the possible peak positions for O=C-O are marked.

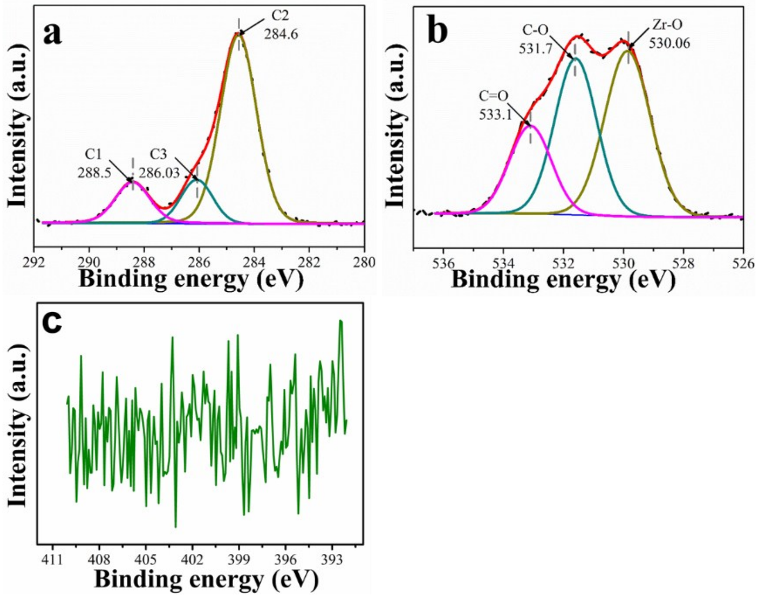

Figure S4 XPS spectra of LAA-4-aerogel, (a) C 1s, (b) O 1s and (c) N 1s.

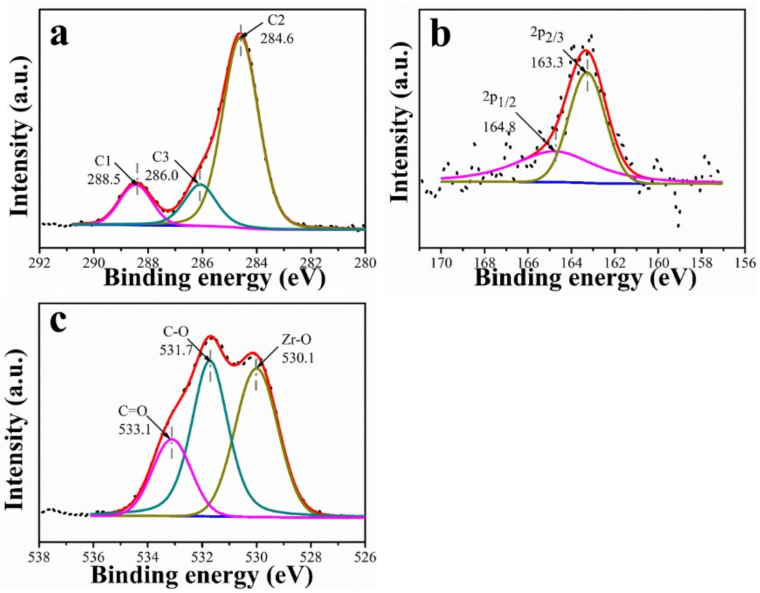

Figure S5 XPS spectra of MSA-8-aerogel, (a) C 1s, (b) S 1s and (c) O 1s.

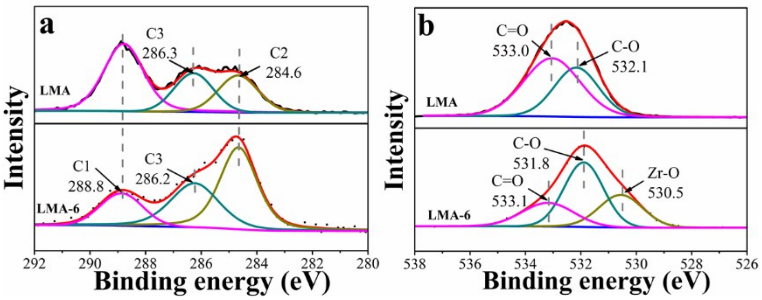

Figure S6 XPS spectra of in organic acid LMA and LMA-6-xerogel, (a) C 1sand (b) O 1s.

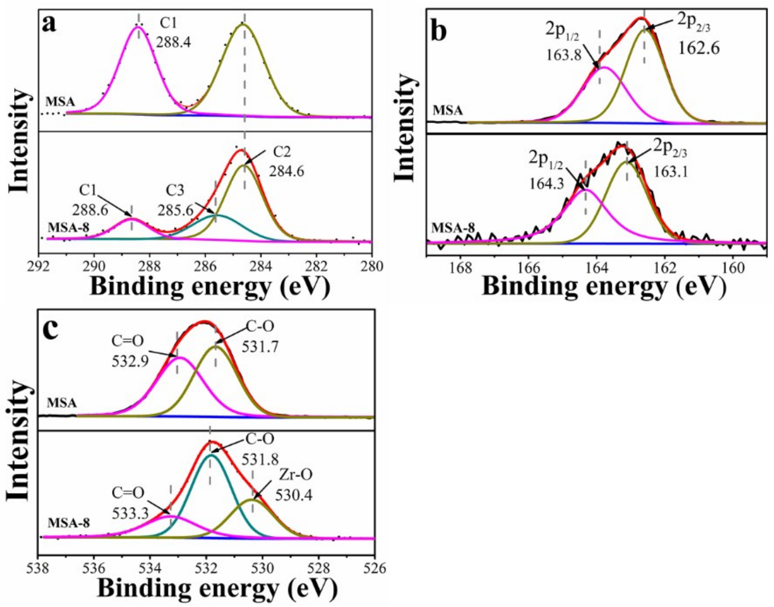

Figure S7 XPS spectra of organic acid MSA and MSA-8-xerogel, (a) C 1s, (b) S 2p and (c) O 1s.

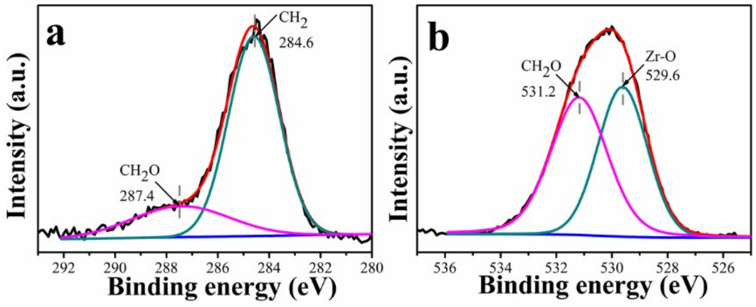

Figure S8 XPS spectra of (a) C 1s and (b) O 1s in PO-8-aerogel.

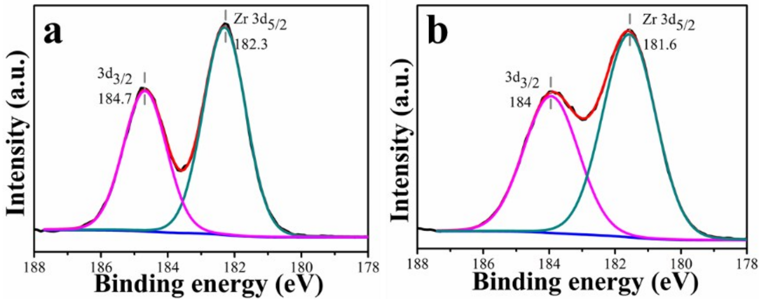

Figure S9 XPS spectra of Zr 3d in LAA-4-aerogel and (b) PO-8-aerogel.

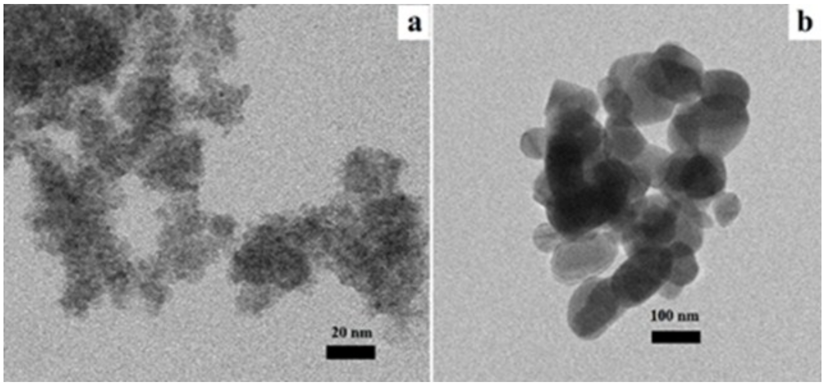

Figure S10 TEM images of MSA-8 aerogel (a) as-prepared and (b) after heat treatment at 1000 °C.

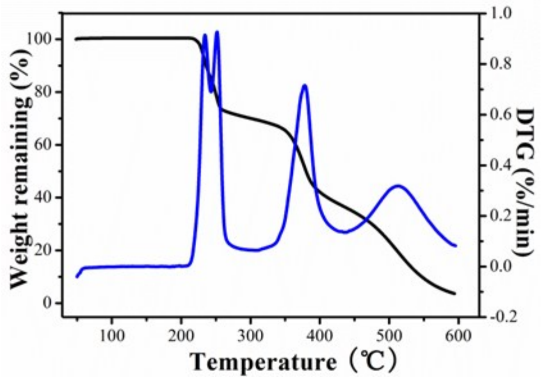

Figure S11 Temperature-dependent weight remaining and the corresponding derivatives of organic acid LAA.
